# Hope

**Published:** 1890-07-05

**Authors:** 


### Words of Consolation.

### HOPE.

One of the greatest blessings of life is hope. It sweetens our cup of suffering, and makes its bitterness and sourness relishing. We should be borne down into gloom and darkness if we could see no end to our sorrows, but hope brightens the scene and shows the way out of our difficulties. The longer we look the clearer becomes our vision, and we end by taking a bright and cheerful view of the future, even though the present seems but doubtful.

In sickness some people lose all hope at once and think they are going to die, which is about the quickest way of making their fears come true. And these are not necessarily the downhearted souls who take a gloomy view of things in general, who "enjoy bad health" as it is called, but those who have previously had no trials to speak of, to whom life has been easy and delightful with good health and spirits. Take away health, and the spirits fall to zero. They cannot think why they of all men should be tormented, and they fret and moan like spoiled children over their adversity. These persons want all the plums of life, and are peevish and angry at anything which gives them husks for food. It is useless to offer them the hope of earthly comforts, which can only restore their cheerfulness for a time, as the joys and fashion of this world pass away. The only hope which lasts and is the anchor of the soul is the firm expectation of heavenly things promised by God to man.

Let us go to the treasure house of the Bible and see what is stored there for our consolation.

Hope was the life of Abraham's faith. Hope led Israel out of Egypt, through the wilderness, through Jordan into Canaan; and will bring us Christians through life, through the waves of this troublesome world, through the dark valley of the shadow of death till we reach the haven of eternal rest in Paradise. Hope converts the world, "by hope are ye saved." The hope of Israel was the Messiah the Lord Jesus Christ, whom St. Paul calls "the hope of the fathers" who looked for His promised coming. Christ is also called our hope, for He is the only One who holds out any foundation on which to build our expectations of earthly good or our prospects for the future life. Hope has a cleansing power, for St. John says "he that hath this hope purifieth himself, even as He is pure."

This Christian hope lightens the eyes and gladdens the heart; it makes religion to shine and be bright and joyful. It prevents faith growing weak and love waxing cold; our very difficulties should but increase our joy, as they show we are in the hand of a wise and loving Father. He sends tribulation which worketh patience, and patience experience, and experience hope, which never shames nor disappoints those who rest upon Him.

The prophet Zechariah, in addressing the Jewish captives who were in Babylon, cries and comforts them in these words, "Turn ye to the stronghold ye prisoners of hope," for they could trust in God's promises of freedom at the end of seventy years. And have not we a Saviour in whom we can trust? Though we be tried and bound with the chain of our sins or are in bondage to Satan, the great enemy of souls, yet our Lord Jesus Christ will free us. He can make us to dwell in safety and possess our souls in patience, for we know that our Redeemer liveth.

